# Polyunsaturated Fatty Acids as Prebiotics: Innovation or Confirmation?

**DOI:** 10.3390/foods11020146

**Published:** 2022-01-06

**Authors:** Emanuele Rinninella, Lara Costantini

**Affiliations:** 1 UOC di Nutrizione Clinica, Dipartimento di Scienze Mediche e Chirurgiche, Fondazione Policlinico Universitario A. Gemelli IRCCS, 00168 Rome, Italy; emanuele.rinninella@unicatt.it; 2 Dipartimento di Medicina e Chirurgia Traslazionale, Università Cattolica del Sacro Cuore, 00168 Rome, Italy; 3 Department of Ecological and Biological Sciences (DEB), Tuscia University, 01100 Viterbo, Italy

**Keywords:** prebiotics, microbiota, polyunsaturated fatty acids, omega-3, omega-6, metabolites

## Abstract

The International Scientific Association for Probiotics and Prebiotics (ISAPP), in its last consensus statement about prebiotics, defined polyunsaturated fatty acids (PUFAs) as “candidate prebiotics” due to a lack of complete scientific evidence. Previous studies have demonstrated the ability of microbiota to metabolize PUFAs, although the role of the resulting metabolites in the host is less known. Recent partial evidence shows that these metabolites can have important health effects in the host, reinforcing the concept of the prebiotic action of PUFAs, despite the data being mostly related to omega-6 linoleic acid and to lactobacilli taxon. However, considering that the symbionts in our gut benefit from the nutritional molecules that we include in our diet, and that bacteria, like all living organisms, cannot benefit from a single nutritional molecule, the concept of the “correct prebiotic diet” should be the new frontier in the field of gut microbiota research.

In 2016, the International Scientific Association for Probiotics and Prebiotics (ISAPP) defined a new consensus statement on the definition and scope of prebiotics [1]. They defined them as: “a substrate that is selectively utilized by host microorganisms conferring a health benefit”. Moreover, the purpose of the ISAPP consensus was to expand the concept of prebiotics to include non-carbohydrate substances. Indeed, their conclusions stated that “currently established prebiotics are carbohydrate-based, but other substances such as polyphenols and polyunsaturated fatty acids converted to respective conjugated fatty acids might fit the updated definition assuming convincing weight of evidence in the target host” and indicated both groups as “candidate prebiotics”. Finally, the definition only includes dietary prebiotics that are not digested by the host but utilized by the microbiota in the gut [1]. The ISAPP defined polyunsaturated fatty acids (PUFAs) as candidate prebiotics because of the lack of the adequate evidence of health benefits for the target host. This is true considering the lack of scientific data in 2016, with only 27 papers published in that year in comparison to the 106 papers published in 2020 (related to the keywords ‘Polyunsaturated Fatty Acids’ and ‘microbiota’, Scopus source). Previous evidence had shown that gut microbes can produce PUFA-derived intermediate metabolites. Indeed, in the paper of Kishino et al. [2], the gut bacterium *Lactobacillus plantarum* was found to produce enzymes involved in the saturation of PUFAs producing the intermediates hydroxy fatty acids, oxo fatty acids, conjugated fatty acids, and partially saturated trans-fatty acids. These intermediates were much higher in the organs of microbiota-colonized mice in comparison to germ-free mice, highlighting the role of microbiota [2]. Subsequently, the same research groups identified the linoleic acid Δ9 hydratase as the enzyme produced by *Lactobacillus plantarum* that catalyzes the first step of PUFA saturation [3], and the same metabolizing activities were also found in the gut bacterium *Lactobacillus acidophilus* [4]. More recently, several microbiota metabolites were found from both omega-3 and omega-6 PUFA precursors, alpha-linolenic acid (ALA, C18:3, omega-3) and linoleic acid (LA, C18:2, omega-6), although the study was conducted on an animal model [5]. So, as described so far, PUFAs already could be considered “a substrate that is selectively utilized by host microorganisms”. This concept is easily conceivable when considering that the food ingested by the host is also, among other compounds, a source of energy for the microorganism inhabiting the gut lumen. Moreover, macronutrients are the staple used to produce the molecules necessary for the activation of specific microbial metabolic pathways. What is worth noting is the mutual interaction that the gut bacteria, and their metabolites, have with the host. So, is there a “health benefit” related to the PUFAs as prebiotics? Some papers have already highlighted the different fatty acids’ local impacts on the gut microbiota, identifying the following order from the least healthy to the healthiest: SFAs < omega-6 PUFAs < MUFAs < omega-3 PUFAs [6]. However, the experimental procedures are very heterogenous and the data are often incomplete, and so this simplification requires further confirmations. Moreover, most of the studies analyzed the taxa variations following diets rich or deficient in PUFAs, and their known health effects. What is missing from the scientific literature is the paucity of metabolite studies that investigate the intestinal microbiota products from PUFAs and their relative effects on the host. The few data about metabolites are mostly collected from the *Lactobacillus* genus and on LA, which represents a small snapshot in comparison to the complexity of the commensals present in the gut microbiota and the totality of PUFAs.

Nonetheless, although in this small niche related to LA metabolism by lactobacilli, the high potential of the microbiota PUFAs metabolism has been demonstrated. Indeed, dietary LA pro-inflammatory potential in the host due to the production of the inflammatory lipid mediators via the arachidonic acid (AA) cascade is well-known. What is noteworthy in the recent paper of Miyamoto and colleagues [5] was the ability of microbiota to mitigate LA pro-inflammatory potential. The authors found that the excessive dietary LA was converted in the microbiota metabolite 10-hydroxy-*cis*-12-octadecenoic acid (HYA), and in physiological conditions, the plasmatic HYA level was higher in comparison to all the other gut microbial PUFA metabolites [5]. Lipids are absorbed in the small intestine, and amazingly *Lactobacillus* and the higher concentration of the metabolite HYA are found in the small intestine, although most of the microbiota normally resides in the colon [5]. Moreover, HYA was found to decrease lipid absorption by increasing peristalsis through the activation of the E-type prostainoid receptor (EP) [5]. So, gut microbiota performs a mutually beneficial action with the host in reducing LA absorption and turning it into a metabolite which is not only less toxic but also beneficial. Indeed, other health-promoting functions have been attributed to HYA and to the other microbiota metabolites from LA. In the same paper, HYA was found to activate G-protein coupled receptor-40 and -120 (GRP40 and GRP120), promoting the secretion of the gut hormone glucagon-like peptide-1 (GLP-1) that stimulates glucose-induced insulin secretion and improves glucose homeostasis [5]. Other previous papers have further shown the positive effects of HYA in improving the intestinal epithelial barrier impairments in dextran sulfate sodium-induced colitis in mice by regulating TNF receptor 2 (TNFR2) expression through the GPR40-MEK-ERK pathway [7]. Similarly anti-inflammatory properties were also systemically found in NC/Nga mice model of atopic dermatitis, where HYA was able to decrease plasma IgE and TNF-α, and increase IgA and the tight-junction protein claudin-1 in the mouse skin [8]; in mouse microglial cells (BV-2), HYA and 10-oxo-trans-11-octadecenoic acid (KetoC) exert anti-inflammatory activities through the inhibition of nitric oxide production and ERK activation by LPS [9]. Indeed, other LA microbiota metabolites can have the same protective activities: Keto C binds GRP120 and activates the NF-κB p65 pathway that suppress the pro-inflammatory cytokines TNF-α, IL-6, and IL-1β in RAW 264.7 mouse macrophagic cells [10]. Moreover, KetoC activates the nuclear factor (erythroid-derived 2)-related factor (Nrf2)-antioxidant response element (ARE) pathway, which enhances cellular antioxidant responses in mice and HepG2 cells [11]. Similarly, 10-oxo-12(Z)-octadecenoic acid (KetoA) is able to activate the transient receptor vanilloid 1 (TRPV1), enhance noradrenalin turnover in adipose tissue ameliorating adiposity and obesity-associated metabolic disorders [12]; also, it activates PPARγ, inducing adipocyte differentiation, increasing adiponectin production and insulin-stimulated glucose uptake in 3T3-L1 murine preadipocytes [13]. Moreover, KetoA has a higher efficiency when reducing triacylglycerol accumulation in HepG2 hepatocytes and significantly decreases the expression of *Srebp-1c*, *Scd-1*, and *Acc2* (i.e., lipogenesis genes) and all risk factors related to cardiovascular diseases (CVDs) in the liver of mice fed a high-sucrose diet (Figure 1) [14]. A comparison between omega-3 and omega-6 PUFAs metabolites showed that omega-6 PUFAs metabolites had greater activity than omega-3 PUFAs metabolites [14]. Miyamoto and colleagues [5] have stated that they found minimal gut microbial omega-3 PUFAs metabolites in physiological conditions and normal chow in mice in comparison to omega-6 PUFAs metabolites. Beyond this information, what is true is the small amount of available information on the effects of ALA-derived microbiota metabolites in comparison to LA- derived microbiota metabolites, such as HYA. It is also true, as happens for the host, that the microbial enzymatic apparatus present for the metabolism of LA is the same for the metabolism of ALA [2]. What is less known is which one, ALA or LA, has a greater affinity for the enzymatic apparatus and if there is a difference between them. For example, in the host, desaturases and elongases have a greater affinity for ALA than LA, but because of the higher intake of dietary LA, more AA from LA than eicosapentaenoic acid (EPA) and docosahexaenoic acid (DHA) from ALA is produced [15]. So, further studies are needed to understand if ALA-derived metabolites are fewer because of enzymatic affinity or due to experimental conditions. Moreover, considering that most of the studies are in animal models, studies in humans are necessary. Conversely, more extensive research was done on the conjugated linoleic acid (CLA) isomers (*trans*-10, *cis*-12-CLA; *cis*-9, *trans*-11-CLA; *trans*-9, *trans*-11-CLA) (Figure 1), because of their intake from ruminant animal origin foods. Although some healthy effects related to their consumption were found [16], it must be considered that the external intake of CLA isomers could be greater than the transformation by the resident microbiota, considering that, as above described, the levels of HYA were higher in comparison to all the other gut microbial PUFA metabolites [5].

Finally, as already mentioned above, the metabolism of few bacteria strains were analyzed. It is also true that enzymes that produce PUFAs metabolites in bacteria belong to the myosin-cross-reactive antigen (MCRA) family proteins that are widely conserved among bacteria [17], so a greater number of bacteria than those now known can metabolize PUFAs and with different affinities between ALA and LA. At the same time, these PUFAs metabolites could play a protective role in inhibiting pathobionts and pathogens engraftment, considering that HYA was found to be able to suppress the growth of *Helicobacter pylori* and *Helicobacter suis* [18]. Lastly, considering that HYA was found to affect the host lipid metabolism through the modulation of peroxisomal β-oxidation activity [19], the metabolic use of these metabolites by the host and the relative effect on health should be explored.

In conclusion, some evidence showed that LA can be a substrate for the *Lactobacillus* genus, which metabolizes it into metabolites, such as HYA, with different health benefits for the host [5,6,7,8,9,10,11,12,13]. Little information is present in the context of humans, and therefore this conclusion cannot be made for all PUFAs. More recently, Vijay and colleagues [20] attempted to prove the prebiotic activities of PUFAs using a different approach. They performed a 6-week dietary intervention on 69 humans with a daily supplementation of 500 mg of omega-3 PUFAs (EPA/DHA) in comparison to 20 g of the well-known prebiotic inulin. Although the microbiota populations underwent different changes between the two supplementations (i.e., *Bifidobacterium* and *Lachnospiraceae* increased in the inulin group, in contrast to the increase in *Coprococcus* and *Bacteroides* in the PUFAs group) both determined significant increases in iso-valerate and iso-butyrate, short-chain fatty acids involved in several healthy actions in the host. They stated that in both supplementations, significant drops in beneficial bacteria were found, suggesting that “[…] the optimal prebiotic supplementation strategies should focus on feeding bacterial communities and requires an understanding of the interdependencies between bacterial strains, rather than the increase of a single carbon source” [20]. So, bacteria, like all living organisms, cannot benefit from a single nutritional molecule, whether it be fibers or fatty acids. The understanding of what can be defined ‘the correct prebiotic diet’ of our symbiotic microbes, and their biochemical-metabolic processes—as the basis of their healthy composition and healthy interactions with the host—should be the new frontier in the field of the gut microbiota research (Figure 2).

## Figures and Tables

**Figure 1:**
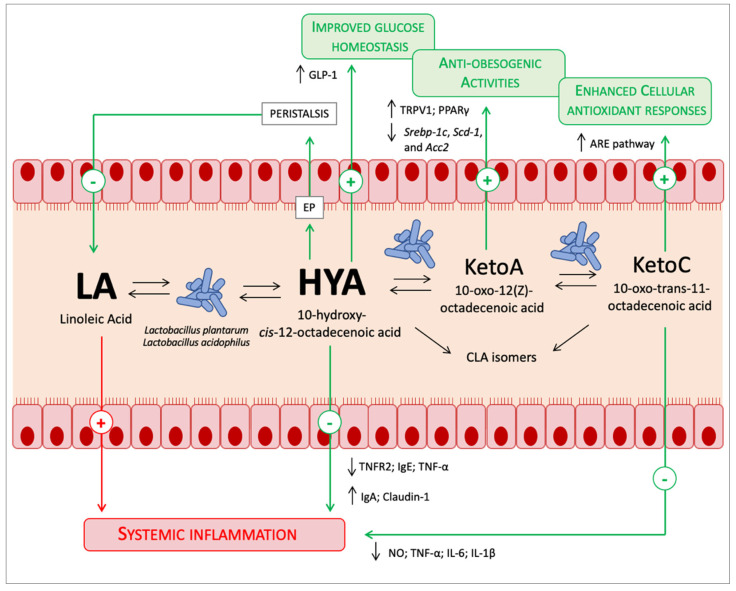
Known prebiotic effects of PUFAs. The font size relative to the PUFAs metabolites is proportional to the quantities found in the gut lumen. See the text for a detailed description.

**Figure 2:**
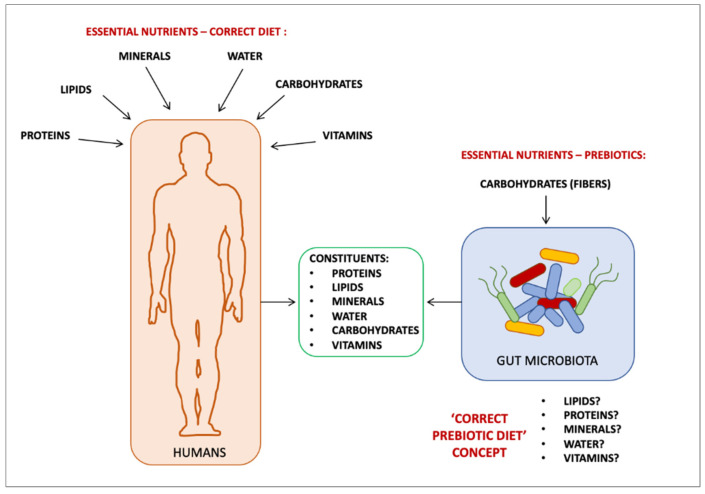
The ‘correct prebiotic diet’ concept.
